# Clinical and functional correlates of parkinsonism in a population-based sample of individuals aged 75 + : the Pietà study

**DOI:** 10.1186/s12883-023-03290-8

**Published:** 2023-07-21

**Authors:** Thiago Cardoso Vale, Francisco Eduardo Costa Cardoso, Danilo Jorge da Silva, Elisa de Paula Franca Resende, Débora Palma Maia, Mauro César Quintão Cunningham, Henrique Cerqueira Guimarães, João Carlos Barbosa Machado, Antônio Lúcio Teixeira, Paulo Caramelli, Maira Tonidandel Barbosa

**Affiliations:** 1 Internal Medicine Department, Faculdade de Medicina, Universidade Federal de Juiz de Fora, Juiz de Fora, MG Brazil; 2 Movement Disorders Unit, Hospital Universitário, Universidade Federal de Juiz de Fora, Juiz de Fora, MG Brazil; 3 Movement Disorders Unit, Hospital das Clínicas, Universidade Federal de Minas Gerais, Belo Horizonte, MG Brazil; 4 Behavioral and Cognitive Neurology Unit, Faculdade de Medicina, Universidade Federal de Minas Gerais, Belo Horizonte, MG Brazil; 5 Faculdade de Medicina, Universidade Federal de Minas Gerais, Belo Horizonte, MG Brazil; 6 Faculdade de Ciências Médicas de Minas Gerais, Belo Horizonte, Brazil

**Keywords:** Parkinsonism, Parkinson’s disease, Dementia, Alzheimer’s disease, Functional decline

## Abstract

**Background:**

Parkinsonism is strongly associated with ageing, and many studies have suggested that parkinsonian signs may affect up to half of older adults and is associated with a wide range of adverse health outcomes. We compared clinical and functional characteristics of oldest-old community-dwelling individuals with parkinsonism (parkinsonian group [PG]) to individuals without parkinsonism (non-parkinsonian group [NPG].

**Methods:**

The Pietà study is a population-based study conducted in Caeté, southeast Brazil, involving 607 individuals aged 75 + years submitted to an extensive clinical evaluation. A subset of 65 PG individuals (61.5% women, median age of 82 years) was compared to 542 NPG individuals (64.8% women, median age of 80 years).

**Results:**

PG individuals had significantly more functional impairment, clinical comorbidities (including number of falls, loss of bladder control and dysphagia) and major depression. Multivariate analysis revealed that older age, higher UPDRSm scores, lower category fluency test (animals/minute) and delayed recall memory scores were associated with PG. This group was also more cognitively impaired, with lower performance than NPG individuals in the Mini-Mental State Examination, category fluency test (animals/minute), clock drawing and in delayed recall (p < 0.001 for all tests). UPDRSm scores were the most contributing factor to cognition that independently explained variability in functionality of the entire sample.

**Conclusion:**

Individuals aged 75 + years with parkinsonism were significantly more clinically and functionally impaired in this population-based sample. Cognitive dysfunction explained most of the loss of functionality in these patients. UPDRS-m scores contributed independently to explain variability in functionality in the whole sample.

**Supplementary Information:**

The online version contains supplementary material available at 10.1186/s12883-023-03290-8.

## Background

The overall disease burden of parkinsonism in the oldest-old population (in developing countries, defined as individuals aged 75 + years) living in the community is still poorly known. Cross-sectional studies [1–3] suggest that parkinsonian signs may affect up to half of older adults and are associated with a wide range of adverse health outcomes, including an increased risk of dementia [4–6] and death [7]. Since parkinsonism is strongly associated with age [8] and the oldest-old population is growing rapidly [9], particularly in developing countries, the syndrome may constitute a major public health problem in the near future.

The incidence of both parkinsonism and Parkinson’s disease (PD) increases with age, particularly after age 80 [10]. However, there is a shortage of reports focusing specifically on this age group. As a consequence, knowledge about the clinical and pathological characteristics of parkinsonism in the oldest-old is still scarce. This aspect is probably based on the etiologic conundrum given the heterogeneity and concomitance of underlying comorbidities and pathologies affecting this age group. In addition, diagnostic accuracy of parkinsonism in the oldest-old is reduced in studies without follow-up evaluations due to insufficient quality of data, from history taking to neuroimaging evaluation.

In Latin America there are only a few epidemiological studies focusing on these individuals [11–13]. In Brazil, a community-based cohort involving 1186 individuals aged 64 + years (the Bambuí study) [14] revealed a crude prevalence of parkinsonism and PD of, respectively, 7200 per 100.000 and 3300 per 100.000 individuals. The overall prevalence of PD increased with age, reaching a peak among individuals older than 84 years.

In the present study, we compared the clinical, cognitive, neuropsychiatric and functional characteristics of community-dwelling individuals aged 75 + years with parkinsonism (parkinsonian group [PG]) to a sample without parkinsonism (non-parkinsonian group [NPG] from a Brazilian population-based study. We also analyzed which variables were independently associated with the outcome of functional decline in both groups.

## Methods

### Study design and target population

This study is a cross-sectional comparative analysis of the available clinical data of 65 PG individuals and 542 NPG individuals from the Pietà study, a community-based survey of brain aging, carried out in Caeté, Southeast Brazil, from January through April, 2008.

Detailed methodology and prevalence data have been described previously [15, 16].

### Clinical, functional and neuropsychiatric evaluations

All subjects underwent a comprehensive functional, clinical, psychiatric and neurological evaluation, including a Global Functional Questionnaire (ranging from no impairment to cannot perform alone the activities of daily living (ADL), such as lying down or raising from the bed; eating; carefulness with appearance; walking on a straight line; bathing; dressing; toileting on time; climbing stairs; taking medicines on time; walking close to home; shopping; preparing meals; cutting finger nails; cleaning the house and driving), the Functional Assessment Staging (FAST) [17], Pfeffer’s Functional Activities Questionnaire (FAQ) [18], the motor section of the Unified PD rating scale (UPDRSm) [19], the Mini International Neuropsychiatric Interview (M.I.N.I) [20], Geriatric Depression Scale (GDS) [21], and a brief cognitive evaluation, consisting of the Mini-Mental State Examination (MMSE) and the Brief Cognitive Screening Battery (BCSB) [22] which includes category fluency test (animals/minute), clock drawing test and the Figure Memory test (FMT, which includes naming, incidental, immediate memory, learning, delayed recall and recognition of 10 drawings).

### Dementia and parkinsonism diagnostic criteria

Diagnosis of cognitive impairment no dementia (CIND) was based on performance on the BCSB and on FAQ scores, according to standard diagnostic criteria [23]. Dementia diagnosis was based on the BCSB and FAQ scores, following the DSM-IV criteria [24]. A reference cut-off on the MMSE was set at the 25th percentile from the epidemiological survey of dementia in a Brazilian community [25]. A cut-off of 5 points on the FAQ was set to define a significant level of functional impairment. Neuropsychological assessment was performed in a subset of individuals and was also used for the diagnosis of CIND and dementia.

Parkinsonism was defined according to the PD Society Brain Research Center of the United Kingdom criteria [26] requiring the presence of bradykinesia and at least one of the following: rest tremor, rigidity, or postural instability not caused by primary visual, vestibular, cerebellar, or proprioceptive dysfunction. We also used a case definition of ‘parkinsonism’ as UPDRSm > 9, since this threshold of parkinsonism has been highly predictive of a clinician's diagnosis of parkinsonism instead of other mimics [27]. A diagnosis of idiopathic PD was established according to the United Kingdom Brain Bank Criteria [26]. Possible PD referred to the presence of two cardinal features while probable PD referred to the presence of three or more cardinal signs (tremor at rest; bradykinesia; rigidity; postural instability). A diagnosis of drug-induced parkinsonism (DIP) was made based on the history of use of anti-dopaminergic drugs for at least six months preceding the onset of symptoms and a tight-close temporal relationship between the drug and the syndrome, along with a previously negative history for the parkinsonian signs. A diagnosis of probable dementia with Lewy Bodies was based on the presence of dementia with at least two of the following core clinical features: spontaneous parkinsonism (concomitant or occurring in up to a year with the dementia syndrome), presence of fluctuating attention and concentration or recurrent well-formed visual hallucinations. A diagnosis of probable vascular parkinsonism was defined by the presence of at least two of the following features, namely, history of repeated strokes with abrupt onset and stepwise progression of parkinsonian features, combined with vascular risk factors and/or neuroimaging markers of a substantial white-matter disease (Fazekas score grade ≥ 3) or strategic infarcts. A diagnosis of probable PD dementia was defined by the fulfillment of criteria for PD combined with parkinsonian signs preceding the development of dementia and an associated global impairment of cognition with functional impairment on the instrumental ADL measured by Pfeffer’s score and FAST staging 4. A diagnosis of parkinsonism-dementia syndrome was established based on the presence of parkinsonism concomitant to at least mild dementia, defined by objective deficit in the BCSB scores according to years of schooling combined with objective functional impairment on the instrumental ADL measured by Pfeffer’s score and FAST staging 4. Among the etiologic possibilities were: mild parkinsonian signs in unspecified demented patients; parkinsonism in the context of possible Alzheimer’s disease; parkinsonism in bedridden advanced demented patients; possible PD-dementia, possible vascular parkinsonism combined with vascular dementia; possible advanced atypical parkinsonism with dementia; possible frontotemporal lobar degeneration with parkinsonism. Confirmation was precluded due to lack of important clinical information on history and follow-up as well as on physical examination. Many patients were from remote rural areas with very limited access to neurologists to record accurate clinical information and physical examination. Finally, unspecified parkinsonism was attributed to patients who did not fulfill the above-mentioned criteria or with insufficient data to reach a possible etiologic classification or with conflicting and concomitant possible etiologies.

### Causes of parkinsonism

The parkinsonian sample was etiologically distributed as such: PD in 19 (29.2%), parkinsonism + dementia syndrome in 19 (29.2%), DIP in eight (12.3%), vascular parkinsonism in four (6.1%) and dementia with Lewy bodies in one (1.5%). In 14 individuals (21.5%), the etiology of parkinsonism could not be determined. A detailed analysis of these causes was previously published [16].

### The non-parkinsonian group

The NPG individuals were syndromic categorized as such: 257 (47.4%) without cognitive impairment, 113 (20.8%) with CIND and 90 (16.6%) with dementia. Eighty-two individuals (15.1% of the sample) could not have their cognitive status defined due to missing data.

### Diagnosis of psychiatric disorders

Neuropsychiatric symptoms such as major depression, psychosis and apathy were screened by a clinical questionnaire and the GDS, and then confirmed through the application of the M.I.N.I, a semi-structured psychiatric interview that permits the diagnosis of mental disorders according to DSM-IV criteria.

### Ethics

The study was approved by the Ethics Committee of the Federal University of Minas Gerais.

### Statistics

All statistical analyses were conducted using the Statistical Package for Social Sciences, version 18 (SPSS, Chicago, IL, USA.). Descriptive statistics of demographic and questionnaire data were provided for continuous [mean and standard deviation (SD), or median, first and third quartiles) and categorical (count and percentage) variables. Normal distribution for continuous numerical variables was verified by the Shapiro–Wilk test. Non-parametric Mann–Whitney test was used in the comparison of the variables without normal distribution, while the T-student test was used in the comparison of variables with normal distribution. Pearson Chi-square or Fisher exact test were used to verify the association hypothesis between the categorical variables of the groups. The adjusted residual values were used as a reference to indicate which classes of variables were associated to a group. We used a p < 0.05 as the value of significance. Variables with p-value < 0.20 were considered for the multivariate logistic regression or the multivariate regression Poisson analysis with robust covariance matrix to verify association of variables to the outcome of, respectively, being in the PG and having a FAQ score above 5 points. Hierarchical linear regression was also used to improve the model’s ability to investigate a moderating effect of different variables on the functionality.

## Results

PG individuals were comprised mostly by women (61.5%), with median age (interquartile range) of 82 (78–87) years and median of 2 (0.25–3.75) years of schooling. NPG individuals were comprised mostly by women (64.8%) with median age of 80 (77–83) years and median of 3 (0–4) years of schooling.

PG individuals were significantly older than those from the NPG. The frequencies of vascular risk factors were not significantly different between the groups. Engagement in physical activities was more common in the NPG (25.0%) compared to the PG (9.7%).

Previous history of stroke was statistically more common in the PG (16.9% vs. 10.7%), as well as the presence of irregular cardiac rhythm suggestive of atrial fibrillation (24.2% vs. 12.5%), but the same did not occur with coronary heart disease. Common clinical comorbidities such as visual, hearing and articular impairment occurred in both groups without statistical difference. However, loss of bladder control, number of falls with fractures and dysphagia were significantly more common in the PG. Table 1 shows the sociodemographic, vascular risk factors and clinical comorbidities data while Table 2 shows the functional data of both groups.

**Table 1:** Sociodemographic and clinical comorbidities among parkinsonian group (PG) and non-parkinsonian group (NPG)

Variable	PG (*N* = 65)	NPG (*N* = 542)	*P*-value
**Sex**^**a**^	**n: 65**	**n: 542**	**0.608**
Female	40 (61.5)	351(64.8)	
Male	25 (38.5)	191(35.2)	
**Age**^**b**^	**n: 65**	**n: 542**	< 0.001*
	82.0 (78.0–87.0)	80.0 (77.0–83.0)	
**Schooling (years)**^**b**^	**n: 65**	**n: 542**	**0.154**
	2.0 (0.25–3.75)	3.0 (0.0–4.0)	
**Arterial hypertension**^**a**^	**n: 65**	**n: 538**	**0.064**
	42 (64.6)	405 (75.3)	
**Diabetes**^**a**^	**n: 65**	**n: 533**	**0.868**
	13 (20.0)	102 (19.1)	
**Dyslipidemia**^**a**^	**n: 65**	**n: 531**	**0.949**
	13 (20.0)	108 (20.3)	
**Osteoarthrosis**^**a**^	**n: 64**	**n: 529**	**0.118**
	14 (21.9)	166 (31.4)	
**Falls with fractures**^**a**^	**n: 65**	**n: 540**	0.008*
	42 (64.6)†	255 (47.2)	
**History of stroke**^**a**^	**n: 65**	**n: 542**	< 0.001*
	11 (16.9)†	58 (10.7)	
**Coronary heart disease**^**c**^	**n: 65**	**n: 539**	**0.511**
	4 (6.2)	22 (4.1)	
**Irregular cardiac rhythm**^**a**^	**n: 62**	**n: 514**	0.011*
	15 (24.2)†	64 (12.5)	
**Carotid bruits**^**c**^	**n: 60**	**n: 508**	**0.571**
	5 (8.3)	31 (6.1)	
**Smoking**^**a**^	**n: 61**	**n: 521**	**0.959**
	18 (29.5)	152 (29.2)	
**Alcohol use**^**a**^	**n: 57**	**n: 489**	**0.053**
	3 (5.3)	71 (14.5)	
**Ankle-brachial- index**^**a**^	**n: 38**	**n: 355**	**0.125**
Normal	20 (52.6)	197 (55.5)	
Arterial calcification	7 (18.4)	30 (8.5)	
Peripheral occlusive disease	11 (28.4)	128 (36.1)	
**Diagnosis of depression**^**a**^	**n: 65**	**n: 539**	0.038*
	21 (32.3)†	113 (21.0)	
**Physical activities**^**a**^	**n**_**:**_** 62**	**n: 520**	0.007*
	6 (9.7)	130 (25.0)†	

Data are presented either as absolute followed by relative frequency (%) or median (1st Quartile-3rd Quartile); n: number of subjects analyzed in the parkinsonian group or in the non-parkinsonian group; ^a^Pearson Chi-Square Test; ^b^Mann-Whitney Test; ^c^Fisher Exact Test; *p-values < 0.05; † adjusted residual value > 1.96

**Table 2:** Functional data of parkinsonian group (PG) and non-parkinsonian group (NPG)

Variable	PG (*N* = 65)	NPG (*N* = 542)	*P*-value
**Impaired bladder control**^**a**^	**n: 63**	**n: 531**	0.006*
	22 (34.9)†	105 (19.8)	
**Dysphagia**^**b**^	**n**_**:**_** 64**	**n**_**:**_** 531**	< 0.001*
	20 (31.2)†	52 (9.8)	
**Visual impairment**^**a**^	**n: 61**	**n: 528**	**0.072**
	28 (45.9)	181 (34.3)	
**Hearing impairment**^**a**^	**n: 60**	**n: 525**	**0.189**
	24 (40.0)	166 (31.6)	
**Lying down or raising from bed**^**a**^	**n: 61**	**n: 512**	< 0.001*
No impairment	24 (39.3)	346 (67.6)†	
Impairment	37 (60.7)†	166 (32.4)	
**Eating**^**b**^	**n: 61**	**n: 517**	< 0.001*
No impairment	44 (72.1)	468 (90.5)†	
Impairment	17 (27.9)†	49 (9.5)	
**Careful with appearance**^**b**^	**n: 60**	**n: 514**	< 0.001*
No impairment	34 (56.7)	448 (87.2)†	
Impairment	26 (43.4)†	66 (12.8)	
**Walking in a straight line**^**a**^	**n: 61**	**n: 509**	< 0.001*
No impairment	27 (44.3)	383 (75.2)†	
Impairment	34 (55.7)†	126 (24.8)	
**Bathing**^**b**^	**n: 61**	**n: 514**	< 0.001*
No impairment	35 (57.4)	441 (85.8)†	
Impairment	26 (42.7)†	73 (14.2)	
**Getting dressed**^**b**^	**n: 61**	**n: 514**	< 0.001*
No impairment	36 (59.0)	449 (87.4)†	
Impairment	25 (41.0)†	65 (12.6)	
**Toileting on time**^**b**^	**n: 61**	**n: 510**	< 0.001*
No impairment	36 (59.0)	458 (89.8)†	
Impairment	25 (40.9)†	52 (10.2)	
**Climbing stairs**^**a**^	**n: 57**	**n: 505**	< 0.001*
No impairment	13 (22.8)	295 (58.4)†	
Impairment	44 (77.3)†	210 (51.6)	
**Taking medicine on time**^**b**^	**n: 58**	**n: 507**	< 0.001*
No impairment	22 (37.8)	395 (77.9)†	
Impairment	36 (62.1)†	112 (22.1)	
**Walking in the neighborhood**^**a**^	**n: 53**	**n: 498**	< 0.001*
No impairment	18 (34.0)	355 (71.3)†	
Impairment	35 (66.0)†	143 (28.7)	
**Shopping**^**b**^	**n: 31**	**n: 386**	< 0.001*
No impairment	12 (38.7)	253 (65.6)†	
Impairment	19 (61.2)†	133 (34.4)	
**Preparing meals**^**b**^	**n: 30**	**n: 397**	< 0.001*
No impairment	14 (46.7)	326 (82.1)†	
Impairment	16 (53.3)†	71 (17.9)	
**Cutting finger nails**^**b**^	**n: 42**	**n: 407**	< 0.001*
No impairment	10 (23.8)	213 (52.3)†	
Impairment	32 (76.2)†	194 (47.7)	
**Driving**^**b**^	**n: 56**	**n: 500**	< 0.001*
No impairment	12 (21.4)	311 (62.2)†	
Impairment	44 (78.6)†	189 (37.8)	
**Cleaning the house**^**b**^	**n: 27**	**n: 350**	< 0.001*
No impairment	5 (18.5)	214 (61.1)†	
Impairment	22 (81.5)†	136 (38.9)	
**Walking**^**b**^	**n: 62**	**n: 520**	< 0.001*
Alone	39 (62.9)	452 (86.9)†	
Need occasional help	7 (11.3)	27 (5.2)	
Need frequent help	11 (17.7)†	26 (5.0)	
Non-ambulatory	5 (8.1)†	15 (2.9)	
**Devices to help in walking**^**b**^	**n: 62**	**n: 510**	< 0.001*
Walking sticks	11 (17.7)†	36 (7.1)	
Walker	2 (3.2)	6 (1.2)	
Wheelchair	4 (6.5)†	9 (1.8)	
Walk without any device	42 (67.7)	449 (88.0)†	
Not ambulatory	3 (4.8)	10 (2.0)	
**Restriction to bed**^**b**^	**n: 62** 5 (8.1)	**n: 520** 20 (3.8)	**0.172**
**FAQ score**^**c**^	**n: 65** 7.0 (1.0–23.0)	**n: 513** 1.0 (0.0–4.0)	< 0.001*
**FAST stage**^**c**^	**n: 62** 4.0 (2.0–8.0)	**n: 501** 0.0 (0.0–2.0)	< 0.001*

Data are presented either as absolute followed by relative frequency (%) or median (1st Quartile-3rd Quartile); n: number of subjects analyzed in the parkinsonian group or in the non-parkinsonian group; ^a^Pearson Chi-Square Test; ^b^Fisher Exact Test; ^c^ Mann–Whitney Test; *p-values < 0.05. † adjusted residual value > 1.96

All functional abilities were significantly altered in the PG when compared to the NPG. Being non-ambulatory and needing frequent help with walking, as well as the use of walking stick or wheelchair, were significantly more associated with parkinsonism. Five (8.1%) PG individuals were not ambulatory and restricted to bed. Median total FAQ score was 7 points and the median FAST stage was 4, both statistically different from the scores of the NPG.

Twenty-one (32.3%) PG individuals presented major depression, in comparison to 113 (21.0%) NPG subjects. The GDS scale score was significantly higher in the PG. Cognitive performance was also significantly more impaired in the PG and the diagnosis of dementia was significantly more frequent in the PG when compared to the NPG. Table 3 displays the cognitive and motor performance and the GDS scores of the groups.

**Table 3:** Motor, cognitive and depressive scale scores in parkinsonian group (PG) and non-parkinsonian group (NPG)

Variable	PG (*N* = 65)	NPG (*N* = 542)	*P*-value
**UPDRSm score**^**a**^	**n: 65** 32.0(21.5–47.0)	**n: 363** 3.0(1.0–8.0)	< 0.001*
**MMSE Score**^**a**^	**n: 59**	**n: 526**	
	18.0(13.0–21.0)	22.0(18.0–25.0)	< 0.001*
**Category fluency score**^**a**^	**n: 59**	**n: 519**	
	7.0(5.0–10.0)	11.0(8.0–14.0)	< 0.001*
**Clock drawing score**^**a**^	**n: 59**	**n: 490**	
	1.0(1.0–4.0)	5.0(2.0–8.0)	< 0.001*
**FMT delay recall score**^**a**^	**n: 59**	**n: 518**	
	4.0(2.0–7.0)	7.0(5.0–8.0)	< 0.001*
**FMT recognition score**^**a**^	**n: 59**	**n: 503**	
	8.0(3.0–10.0)	10.0 (9.0–10.0)	< 0.001*
**Cognitive status**^**b**^	**n**_**:**_** 65**	**n**_**:**_** 542**	< 0.001*
1. Cognitively healthy	11 (16.9%)	257 (47.4%)†	
2. CIND	17 (26.1%)	113 (20.8%)	
3. Dementia	37 (56.9%)†	90 (16.6%)	
4. Undetermined	0 (0.0%)	82 (15.1%)†	
**GDS total score**^**a**^	**n: 52** 6.0(4.0–9.0)	**n: 502** 3.0(1.0–5.0)	< 0.001*

Data are presented either as absolute followed by relative frequency (%) or median (1st Quartile-3rd Quartile); n: number of subjects analyzed in the parkinsonian group or in the non-parkinsonian group; UPDRSm: Unified Parkinson’s disease rating scale-Part III (motor); MMSE: Mini-Mental State Examination; FMT: Figure Memory Test; CIND: cognitive impairment no-dementia; GDS: Geriatric Depression Scale; ^a^Mann-Whitney Test; ^b^Pearson Chi-Square Test; *p-value < 0.05 († adjusted residual value > 1.96)

Given the high number of potentially confounding variables and the significant age difference between the two groups, a multivariate logistic regression analysis was used to detect the variables independently associated with the PG (Table 4). Increased age and UPDRSm scores as well as lower FMT delayed recall and category fluency scores were independently associated with being in the PG.

**Table 4:** Multivariate logistic regression analysis with the outcome being in the PG

Variable	Β	*p*-value	OR (95% CI)
**Age**	0.092	0.003	1.096 (1.031–1.166)
**UPDRSm score**	0.080	< 0.001	1.084 (1.054–1.114)
**FMT delayed recall score**	- 0.229	0.002	0.796 (0.656–0.965)
**Category fluency score**	- 0.156	0.002	0.856 (0.774–0.946)

*PG* Parkinsonian group; Β: Logistic regression model coefficient; OR: Odds-ratio; CI: confidence interval; UPDRSm: Unified Parkinson’s disease rating scale-Part III (motor); FMT: Figure Memory Test; Hosmer Lemeshow = 0.891; R^2^ Nagelkerke = 0.391

Multivariate regression Poisson analysis was used to detect the variables independently associated with functional decline (FAQ > 5 points). Fifteen variables (age; sex; schooling years; being in the PG; depression; previous history of stroke; dysphagia; UPDRSm; MMSE; category fluency; clock drawing; delayed recall and recognition scores of the FMT; and GDS score) showed significant associations with functional decline and were used for the analysis (Supplement 1). Being in the PG and having lower scores in the delayed recall of the FMT were independently associated with functional decline in this regression model (Supplement 2). Prevalence of parkinsonism in individuals with functional decline was 25.6% higher (95% CI: 8.8% to 45.1%) than in individuals without functional decline. A one-point reduction in the FMT delayed recall score increased in 8.5% (95% CI: 5.6 to 11.4%) the frequency of functional decline.

When the PG was evaluated separately from the NPG (Supplement 3), presence of dysphagia (p = 0.032; PR 1.383 [95% CI: 1.028–1.859]) and a low delayed recall score in the FMT (p = 0.001; PR 0.919 [95% CI: 0.874–0.967]) were independently associated with functional decline in the regression analysis. Frequency of dysphagia in individuals from the PG with FAQ > 5 was 1.38 higher than in individuals from the PG with FAQ ≤ 5. A one-point reduction in the FMT delayed recall scores increased in 8.8% (95% CI: 3.4–14.4%) the frequency of individuals from the PG with FAQ > 5.

When the NPG was evaluated separately (Supplement 4), UPDRSm scores (p = 0.001; PR 1.023 [95% CI: 1.014–1.032]) was the only variable independently associated with functional decline in the regression analysis. A one-point increase in the UPDRSm score was associated with a 2.3% (95% CI: 1.4–3.2%) higher chance of manifesting functional decline with FAQ > 5.

Given the independent association of UPDRSm scores with functional decline in the NPG seen in the regression analysis and the confounding presence of cognitive variables determining functional decline in the PG group, we decided to carry out a hierarchical linear regression to moderate the effect of the variables. The hierarchical model **(**Table 5**)** defined one block of variables related to cognition and added variables not related to cognition such as the presence of dysphagia and the UPDRSm score to analyze the variability in the functionality measured by FAQ scale points. Cognition was indeed the most contributing factor associated with loss of functionality, but UPDRSm scores resulted in an added burden to patient’s functionality. UPDRSm scores contribution is 3.8%, a small but significant percentual. UPDRSm score explains 66.3% of the variability found in the FAQ scores in the whole sample.

**Table 5:** Hierarchical linear regression models in the whole sample

Model	R	R^2^	R^2^ adjusted	R^2^ change	F
1	0.782	0.612	0.607	0.612	120.104
2	0.794	0.631	0.625	0.019	19.854
3	0.795	0.632	0.625	0.001	0.562
4	0.818	0.670	0.663	0.038	43.849

Model 1: presence of dementia, schooling years, picture drawing memory test’s delayed recall score, picture drawing memory test’s recognition score, Mini-Mental State Examination; Model 2 = Model 1 + dysphagia; Model 3 = Model 2 + being in the parkinsonian group; Model 4 = Model 3 + Unified Parkinson’s disease rating scale motor scores

## Discussion

Our findings indicate that parkinsonism in this oldest-old population-based sample was associated with significant clinical, cognitive and psychiatric comorbidities. Older age, higher UPDRSm scores, lower delayed recall on the FMT and lower category fluency scores were independently linked to parkinsonism in the regression analysis. Moreover, being in the PG and having lower FMT delayed recall scores were independently associated with functional decline in the whole sample. When the PG sample was separately analyzed, presence of dysphagia and lower FMT delayed recall scores were independently associated with functional decline in the regression model. Higher UPDRSm scores in the NPG sample were also independently associated with functional decline. In the hierarchical linear regression analysis, UPDRSm scores significantly contributed to cognition in the functional decline of the whole sample. These comorbidities lead to an important decline in functional performance, which may increase caregiver burden and decrease quality of life.

Indeed, in a longitudinal, community-based study which enrolled 455 individuals 65 + years from East Boston in the US (mean age of 80.5 ± 7.6 years), the presence of parkinsonism strongly predicted progressive disability and had a marked aging effect on disability level [28]. At baseline, participants with parkinsonism presented higher levels of disability than those without parkinsonism. After adjustment for age, sex, and comorbidity in the longitudinal analysis, parkinsonism was also significantly associated with disability. Age is of utmost importance in disability as shown in a single center, case–control study comparing 60 old-old (≥ 85 years of age) to 92 young-old (aged 60–75 years) PD patients, matched for disease duration. The old-old PD patients predominantly had significantly greater postural instability, gait disturbance, higher UPDRSm scores and more advanced disease stage with higher scores on the non-motor symptoms questionnaire [29].

Clinical cohort studies of parkinsonian signs in persons without PD have found that parkinsonism is associated with increased risk of falls [30], morbidity and nursing home placement [31]. According to Buchanan et al*.* [32], residents of nursing homes with PD who were aged 79.7 years on average at admission tended to be physically dependent and cognitively impaired. They found a high prevalence of dementia and depression, which significantly impacted their functionality and quality of life [33]. Another study in a community setting showed that parkinsonian signs were associated with a two-fold increased risk of death [7, 34].

The PG of our study was significantly more functionally impaired when compared to NPG. Functions that require motor control were expectedly more impaired in the PG, but functional decline also occurred in other abilities, such as careful with appearance, toileting on time, taking medicine on time and preparing meals. FAQ scores and a mean FAST staging of 4 points, significantly higher in PG individuals, reveal at least a mild dementia stage, requiring assistance in instrumental activities. Diagnosis of dementia was significantly more frequent in the PG. Our study showed that cognitive dysfunction is the most important factor predicting functional decline in this oldest-old population and the UPDRSm scores can contribute independently to the functional decline.

Disability is a significant and costly public health problem [35], frequently attributed to multiple comorbidities. In the Framingham study [36], older participants were evaluated for the effects of specific medical conditions on functional limitations. Stroke, depressive symptomatology, hip fracture, knee osteoarthritis and heart disease accounted for most of the observed disability. In our sample, stroke and depression were significantly linked to parkinsonism and these concomitant conditions might have contributed to worse functional status as compared to the NPG. In fact, among the parkinsonian group, almost a third of patients presented with muscle weakness, either in the form of motor deficits secondary to stroke or a generalized weakness that may correspond to the elderly frailty [16]. In a Japanese community-dwelling study involving 613 persons aged 65 + years, GDS scores were significantly higher in the mild parkinsonian signs group as compared to the NPG [37]. Results from the EURODEP collaboration study showed that depression is highly common in community-dwelling older adults with parkinsonism, even among those without functional disability [38]. To make matters worse, depressed individuals derived from our study sample presented several cognitive and functional deficits [39].

Our study shows that the PG performed significantly poorer than the NPG in many cognitive tests, revealing a frequent combination of dementia in this group, with a frequency that was significantly higher than in the NPG. The two groups were matched for education and for the presence of visual and hearing impairment, two relevant features that could interfere with cognitive evaluation. Dementia was diagnosed in 37 (56.9%) of PG individuals, being PD dementia, Alzheimer’s disease followed by vascular dementia the most prevalent among the identified etiologies [16]. In the TREND study [40], among 480 neurologically healthy individuals aged between 50 and 80 years, 52 (11%) had mild parkinsonian signs and were evaluated longitudinally for their cognitive performance. Participants were not significantly different from controls in relation to depressive symptomatology and vascular risk factors. However, they showed worse cognitive performance compared with controls and lower plasma Aβ_1–42_ levels in the baseline and longitudinal evaluations. These results suggest that individuals with persistent mild parkinsonian signs might be in a prodromal phase of dementia. In our cohort, diagnosis of depression was significantly more common in the PG, what might have led to poorer cognitive performance. It is also important to state that PG individuals were older than NPG subjects, what is actually expected among this disease group.

Except for a previous history of stroke and higher prevalence of irregular cardiac rhythm, our PG individuals did not differ from the NPG when vascular risk factors were analyzed. Likewise, no association has been found between these vascular factors with parkinsonism and functional loss. However, previous studies have linked the cerebral small-vessel disease, which has the hypertensive microangiopathy as one major etiology, to mild parkinsonian signs in older individuals [41, 42]. Indeed, cerebral small-vessel disease is a leading cause of stroke, cognitive impairment, gait and balance disturbances, and disability in older subjects [43]. This is conspicuously seen in patients with vascular parkinsonism, who present arterial hypertension as a frequent risk factor and are more cognitively impaired and frequently fulfilling diagnostic criteria for concomitant vascular dementia [44]. Resende et al. [45], in a neuroimaging analysis of individuals from the Pietà study, involving 177 subjects (112 cognitively healthy; 36 with CIND and 29 with dementia), found a high prevalence of small-vessel disease with severe white matter lesions associated with hypertension and cognitive impairment. This high prevalence occurred even in cognitively healthy subjects. Although hypertension was common in our PG subjects, it did not differ from the NPG and its role in the contribution of cerebral small-vessel disease was beyond the scope of the present article [46].

We acknowledge the existence of several limitations related to the methodology and the sample of our study. Individuals were selected from a population-based study, with a rather limited sample size and not overall representative of the Brazilian community-dwelling oldest-old population, giving the abundant regional differences that exists in Brazil. Likewise, and among our main limitations, were the clinical grounds on which our etiologic diagnosis was made, without laboratory and neuroimaging evaluations. We encountered a high number of unspecified parkinsonism and dementia and some were categorized as parkinsonism-dementia syndrome, which encounters many possible etiologies.

## Conclusions

Brazilian individuals aged 75 + with parkinsonism derived from a population-based sample had significantly more clinical (previous history of stroke, irregular cardiac rhythm, loss of bladder control, higher number of falls with fractures and dysphagia), cognitive (higher rates of dementia) and psychiatric comorbidities (depression). They were more functionally impaired, mainly due to cognitive dysfunction, when compared to controls. To access the variables independently associated with functional decline in the whole sample, we used a hierarchical linear regression analysis which showed that UPDRSm scores contributed independently to explain variability in functionality and, together with cognitive impairment, contributed significantly with functional decline of the whole sample. This work can help benefit the community as it shows that an earlier identification of parkinsonism and parkinsonian signs may help in managing these patients and in reducing the impact of the motor symptoms on functionality.

## Supplementary Information

**Additional  file 1.** 

## Data Availability

The datasets used and/or analysed during the current study are available from the corresponding author on reasonable request.

## Abbreviations

PD: Parkinson’s disease
PG: Parkinsonian group
DIP: Drug-induced parkinsonism
NPG: Non-parkinsonian group
FAST: Functional Assessment Staging
FAQ: Functional Activities Questionnaire
UPDRSm: The motor section of the Unified Parkinson’s disease rating scale
MINI: Mini International Neuropsychiatric Interview
GDS: Geriatric depression scale
BCSB: Brief cognitive screening battery
MEEM: Mini-mental state examination
FMT: Figure memory test
CIND: Cognitive impairment non-dementia
DSM-IV: Disease Statistics Manual 4^th^ Edition
SD: Standard deviation
