# Supplementary material for: Clinical and functional correlates of parkinsonism in a population-based sample of individuals aged 75 + : the Pietà study
Source: BMC Neurol. 2023 Jul 21;23:276. doi: 10.1186/s12883-023-03290-8 (PMC10360246; doi:10.1186/s12883-023-03290-8)
Supplement: Supplementary file 1 — Additional file 1. [file 12883_2023_3290_MOESM1_ESM.docx]

**Appendix 1: Descriptive data and analysis of the association between variables and the FAQ’s score**

| Variable | FAQ > 5  (N_1_=154) | FAQ ≤ 5  (N_2_=422) | P-value |
| --- | --- | --- | --- |
| Group^1^  PG  NPG | 36 (23.4)†  118 (76.6) | 27 (6.4)  395 (93.6)† | <0.001* |
| Sex^1^  Male  Female | 47 (30.5)  107 (69.5) | 154 (36.5)  268 (63.5) | 0.183* |
| Age^2^ | 82.5 (78.8-87.0) | 79.0 (76.0-83.0) | < 0.001* |
| Schooling (years)^2^  (N_1_: 148; N_2_: 413) | 2.0 (0.0-4.0) | 3.0 (1.0-4.0) | < 0.001* |
| History of stroke^1^  Yes  No | 34 (22.1)†  120 (77.9) | 31 (7.3)  391 (92.7)† | <0.001* |
| History of falls^1^  Yes  No | 78 (51.0)  75 (49.0) | 207 (49.1)  215 (50.9) | 0.683 |
| History of heart attacks^1^  Yes  No | 6 (3.9)  146 (96.1) | 17 (4.0)  404 (96.0) | 0.961 |
| Presence of dysphagia^1^  Yes  No | 34 (22.7)†  116 (77.3) | 37 (8.9)  380 (91.1)† | <0.001* |
| Diagnosis of depression^1^  Yes  No | 45 (29.2)†  109 (70.8) | 80 (19.0)  342 (81.0)† | 0.008* |
| UPDRSm score^2^  (N_1_: 103; N_2_: 306) | 16.0 (6.0-33.0) | 3.0 (1.0-8.0) | <0.001* |
| MMSE^2^  (N_1_: 138; N_2_: 421)  GDS^2^:  (N_1_: 112; N_2_: 416) | 15.0 (12.75-19.0)  4.0 (2.0-7.0) | 23 (20.0-26.0)  2.0 (1.0-4.0) | <0.001*  <0.001* |
| Category fluency test score^2^  (N_1_: 131; N_2_: 420) | 7.0 (5.0-10.0) | 12.0 (9.0-15.0) | <0.001* |
| Clock drawing test score^2^  (N_1_: 124; N_2_: 399) | 1.5 (1.0-3.0) | 5.0 (3.0-9.0) | <0.001* |
| Delayed recall FMT score^2^  (N_1_: 130; N_2_: 420) | 4.0 (0.0-6.0) | 7.0 (6.0-8.0) | <0.001* |
| Recognition FMT score^2^  (N_1_: 123; N_2_: 413) | 8.0 (4.0-9.0) | 10.0 (9.0-10.0) | <0.001* |

Data are presented as absolute followed by relative frequency (%) or median (1st Quartile-3rd Quartile); N_1_: number of subjects with FAQ’s score > 5; N_2:_ number of subjects with FAQ’s score ≤ 5; FAQ: Functional Activities Questionnaire; PG: Parkinsonian group; NPG: Non-parkinsonian group; UPDRSm: Unified Parkinson’s disease rating scale-Part III (motor); MMSE: Mini-Mental State Examination; GDS: Geriatric Depression Scale; FMT: Figure Memory Test; ^1^Pearson Chi-Square Test; ^2^Mann-Whitney test; *p-value<0.20 († adjusted residual value>1.96).

**Appendix 2: Multivariate regression Poisson analysis with robust covariance matrix with outcome FAQ’s scores in the PG and NPG.**

| Variable | p-value | PR (95% CI) |
| --- | --- | --- |
| Being in the PG | 0.002 | \| 1.256 (1.088-1.451) \| \| --- \| |
| FMT delayed recall score | <0.001 | 0.922 (0.898-0.947) |

PR: Prevalence ratio; CI: confidence interval; PG: Parkinsonian group; FMT: Figure Memory Test

**Appendix 3: Descriptive data and analysis of the association between variables and the FAQ’s score for the parkinsonian group**

| Variable | FAQ > 5  (N_1_=36) | FAQ ≤ 5  (N_2_= 27) | P-value |
| --- | --- | --- | --- |
| Sex^1^  Male  Female | 15 (41.7)  21 (58.3) | 9 (33.3)  18 (66.7) | 0.500 |
| Age^2^  (N_1_=36; N_2_=27) | 83 (78-88) | 81 (78-85) | 0.354 |
| Schooling (years)^2^  (N_1_=36; N_2_=27) | 2.0 (0.0-3.0) | 2.0 (0.0-4.0) | 0.492 |
| History of stroke^3^  Yes  No | 8 (22.2)  28 (77.8) | 3 (11.1)  24 (88.9) | 0.326 |
| History of heart attack^3^  Yes  No | 3 (8.3)  33 (91.7) | 1 (3.7)  26 (96.3) | 0.629 |
| History of falls^1^  Yes  No | 25 (69.4)  11 (30.6) | 16 (59.3)  11 (40.7) | 0.401 |
| Presence of dysphagia^1^  Yes  No | 16 (44.4)†  20 (55.6) | 4 (15.4)  22 (84.6)† | 0.016* |
| UPDRSm^4^  (N_1_: 36; N_2_: 27) | 38.11 (±15.65) | 27.75 (±16.21) | 0.006* |
| MMSE^2^  (N_1_: 30; N_2_: 27) | 14.0 (9.25-15.75) | 21.0 (19.0-23.0) | <0.001* |
| GDS^2^:  (N_1_: 23; N_2_: 27) | 6.0 (4.0-8.0) | 7.0 (3.0-9.0) | <0.001* |
| Category fluency score^2^  (N_1_: 30; N_2_: 27) | 6.0 (4.0-8.25) | 9.0 (7.0-12.0) | 0.001* |
| Clock drawing test score^2^  (N_1_: 30; N_2_: 27) | 1.0 (1.0-3.2) | 4.0 (1.0-6.0) | 0.294 |
| FMT delayed recall score^2^  (N_1_: 30; N_2_: 27) | 3.0 (0.0-4.2) | 6.0 (4.0-7.0) | < 0.001* |
| FMT recognition score^2^  (N_1_: 30; N_2_: 27) | 5.0 (1.7-9.0) | 10.0 (7.0-10.0) | 0.001* |

Data are presented as absolute followed by relative frequency (%) or median (1st Quartile-3rd Quartile); N_1_: number of subjects with FAQ’s scores > 5; N_2_: number of subjects with FAQ’s scores ≤ 5; FAQ: Functional Activities Questionnaire; PG: Parkinsonian group; NPG: Non-parkinsonian group; UPDRSm: Unified Parkinson’s disease rating scale-Part III (motor); MMSE: Mini-Mental State Examination; GDS: Geriatric Depression Scale; FMT: Figure Memory Test; ^1^Pearson Chi-Square Test; ^2^ Mann-Whitney test; ^3^Fisher Exact Test; ^4^T-student test; *p-value<0.20 († adjusted residual value>1.96).

**Appendix 4: Descriptive data and analysis of the association between variables and the FAQ’s score for the non-parkinsonian group**

| Variable | FAQ > 5  (N_1_=118) | FAQ ≤ 5  (N_2_= 395) | P-value |
| --- | --- | --- | --- |
| Sex^1^  Male  Female | 32 (27.1)  86 (72.9) | 145 (36.7)  250 (63.3) | 0.054* |
| Age^2^  (N_1_=118; N_2_=395) | 82.5 (79.0-86.2) | 80.0 (77.0-83.0) | < 0.001* |
| Schooling (years)^2^  (N_1_=112; N_2_=386) | 2.0 (0.0-4.0) | 3.0 (1.0-4.0) | < 0.001* |
| Diagnosis of depression^1^  Yes  No | 31 (26.3)†  87 (73.7) | 74 (18.7)  321 (81.3)† | 0.075* |
| History of stroke^1^  Yes  No | 26 (22.0)†  92 (78.0) | 28 (7.1)  367 (92.9)† | <0.001* |
| History of heart attack^1^  Yes  No | 3 (2.6)  113 (97.4) | 16 (4.1)  378 (95.9) | 0.586 |
| History of falls^1^  Yes  No | 53 (45.3)  64 (54.7) | 191 (48.4)  204 (51.6) | 0.561 |
| Presence of dysphagia^1^  Yes  No | 18 (15.8)  96 (84.2) | 33 (8.4)  358 (91.6) | 0.022 |
| UPDRSm^2^  (N_1_:67; N_2_: 279) | 8.0 (3.5-16.0) | 2.0 (0.0-6.0) | <0.001* |
| MMSE^2^  (N_1_: 108; N_2_: 394) | 16.0 (13.0-19.0) | 23.0 (20.0-26.0) | <0.001* |
| GDS^2^:  (N_1_: 89; N_2_: 389) | 4.0 (2.0-7.0) | 2.0 (1.0-4.0) | <0.001* |
| Category fluency score^2^  (N_1_: 101; N_2_: 393) | 7.0 (5.0-10.0) | 12.0 (9.0-15.0) | <0.001* |
| Clock drawing test score^2^  (N_1_: 94; N_2_: 372) | 2.0 (1.0-3.2) | 5.0 (3.0-9.0) | <0.001* |
| FMT delayed recall score^2^  (N_1_: 100; N_2_: 393) | 4.0 (1.0-6.0) | 7.0 (6.0-8.0) | < 0.001* |
| FMT recognition score^2^  (N_1_: 93; N_2_: 386) | 8.0 (5.0-9.0) | 10.0 (9.0-10.0) | <0.001* |

Data are presented as absolute followed by relative frequency (%) or median (1st Quartile-3rd Quartile); N_1_: number of subjects with FAQ’s scores > 5; N_2_: number of subjects with FAQ’s scores ≤ 5; FAQ: Functional Activities Questionnaire; UPDRSm: Unified Parkinson’s disease rating scale-Part III (motor); MMSE: Mini-Mental State Examination; GDS: Geriatric Depression Scale; FMT: Figure Memory Test; ^1^Pearson Chi-Square test; ^2^Mann-Whitney test; ^3^Fisher exact test; *p-value<0.20 († adjusted residual value>1.96).
